# Trazodone effectiveness in depression (TED): a comparative evaluation of effect sizes trazodone extended release and SSRIs in the treatment of major depressive disorder

**DOI:** 10.3389/fphar.2026.1739359

**Published:** 2026-04-07

**Authors:** Dominika Dudek, Adrian Andrzej Chrobak, Karolina Podkowa, Anna Julia Krupa, Aleksandra Gorostowicz, Rafał Jaeschke, Andrzej Juryk, Marcin Siwek

**Affiliations:** 1 Department of Adult Psychiatry, Jagiellonian University Medical College, Cracow, Poland; 2 Department of Pathophysiology, Jagiellonian University Medical College, Cracow, Poland; 3 Department of Biological and Community Psychiatry, Jagiellonian University Medical College, Cracow, Poland

**Keywords:** effect size, Major depressive disorder, MDD, SSRIs, trazodone

## Abstract

**Introduction:**

Major depressive disorder (MDD) constitutes a significant global mental health concern. Although selective serotonin reuptake inhibitors (SSRIs) are first-line treatment, their effectiveness may be limited by adverse effects including anhedonia, emotional blunting, sleep disturbances, and sexual dysfunction. Trazodone, a serotonin antagonist and reuptake inhibitor, offers a more favorable tolerability profile, particularly in its extended-release (XR) formulation. Previous studies within the trazodone effectiveness in depression (TED) project demonstrated more pronounced improvements with trazodone XR compared to SSRIs in reducing depressive, anxiety, and insomnia symptoms. The present analysis extends these findings by comparing trazodone XR with SSRIs and quantifying the extent and clinical importance of treatment outcomes through effect size estimates.

**Methods:**

This single-center, non-randomized, open-label, 12-week naturalistic study—conducted as part of the TED project—included adults aged 18–65 diagnosed with MDD. Symptom severity and outcomes were assessed at baseline and weeks 2, 4, 8, and 12 using validated clinician- and self-rated scales. Cohen’s d quantified the magnitude and clinical relevance of differences between trazodone XR and SSRIs.

**Results:**

Effect-size analyses demonstrated consistently greater and earlier improvements with trazodone XR versus SSRIs across all measures. In both self-rated (QIDS-SR) and clinician-rated (QIDS-CR; MADRS) scales assessing depressive symptoms, trazodone XR showed larger effect sizes from week 4, with further increases through week 12. Similar patterns were observed for anhedonia (SHAPS), anxiety (HAM-A) and insomnia (AIS), where trazodone XR produced greater and progressively increasing effect sizes, while SSRIs reached a plateau. These findings indicate a more robust and sustained therapeutic impact of trazodone XR, reflected by consistently higher effect-size magnitudes across domains.

**Discussion:**

Trazodone XR demonstrated greater and progressively increasing effect sizes compared with SSRIs, indicating a more sustained antidepressant response over 12 weeks. This trajectory, marked by continued symptom reduction without a linear response pattern, suggests a cumulative therapeutic effect potentially attributable to trazodone’s multimodal serotonergic mechanism and favorable pharmacokinetics. By concurrently addressing mood, anxiety, and sleep-related domains, trazodone XR appears to facilitate both symptomatic improvement and broader functional stabilization. These findings highlight the need for randomized, controlled investigations to further elucidate its comparative efficacy and real-world relevance.

## 1 Introduction

Major depressive disorder (MDD) is recognized as a significant global mental health concern affecting roughly 4% of the global population; the discrepancy between the efficacy of pharmacotherapy demonstrated in tightly controlled clinical trials and its insufficient performance observed in patients highlights the necessity of evaluating its effectiveness under real-world practice conditions (Ormel et al., 2022; McIntyre and Jain, 2024; World Health Organization, 2025).

Pharmacological interventions remain the primary treatment modality for this insidious disorder, with selective serotonin reuptake inhibitors (SSRIs) representing the most frequently prescribed class of antidepressants internationally, alleviating depressive symptoms by modulating serotonin (5-hydroxytryptamine, 5-HT) transmission (Elmarasi and Fuehrlein, 2024; Peano et al., 2025). Despite their favorable safety profile, the use of SSRIs may be associated with an increased risk of nausea, insomnia, emotional blunting, sexual dysfunction, sweating, dry mouth, diarrhea, dizziness, and asthenia (Walker, 2013; Goodwin et al., 2017; Gosmann et al., 2023). It is worth emphasizing that specific SSRIs-associated adverse effects exert a disproportionately negative impact on treatment adherence. These include, in particular, residual anhedonia, emotional blunting, sleep disturbances, and sexual dysfunction, and are frequently cited by patients as key reasons for poor compliance and premature discontinuation of pharmacotherapy (Rothmore, 2020; Ma et al., 2021; Zhou et al., 2023; Wu et al., 2025). Anhedonia (which exhibits phenotypical overlap with emotional blunting) constitutes a core dimension of MDD, often associated with more severe symptoms, elevated suicide risk, and limited response to standard antidepressant treatments (Ma et al., 2021; Wu et al., 2025). It has been delineated as a distinct depressive endotype characterized by impaired reward processing; however, an increasing number of studies demonstrate that SSRIs exhibit only limited efficacy in ameliorating this symptom (Serretti, 2025; Wu et al., 2025). Sleep disturbances are common consequences of antidepressant treatment and may substantially impair both therapeutic efficacy and overall treatment tolerability. Most SSRIs have been associated with a significantly increased incidence of treatment-emergent somnolence and insomnia compared to placebo (Zhou et al., 2023). Sexual dysfunction is consistently observed during SSRIs treatment, affecting a substantial proportion of patients—up to 80%—and are frequently implicated in non-adherence and treatment dropout (Rothmore, 2020).

Taken together SSRIs are effective in diminishing the overall severity of depressive symptoms; however, it is critical to emphasize that the aforementioned adverse effects, although often under-recognized in routine clinical assessments, are consistently reported by patients as particularly burdensome and have been strongly associated with reduced treatment satisfaction, poor adherence, and premature treatment discontinuation (Cipriani et al., 2018).

In this context, trazodone has garnered attention due to a more favorable profile with respect to anxiety, sleep, and sexual adverse effects, with recent findings suggesting a comparatively reduced risk of these complications relative to SSRIs (Fagiolini et al., 2023). Trazodone, another pharmacological agent that modulates the serotonergic system is a multifunctional psychotropic drug with dose-dependent actions. At lower doses (25–150 mg), it primarily exerts sleep promoting effects, mediated through high-affinity antagonism at 5-HT_2A_, H_1_ (histaminergic), and α_1_ (adrenergic) receptors. These properties underlie its sedative action and are commonly exploited in the treatment of insomnia (Fagiolini et al., 2023). In contrast, higher doses (150–600 mg) are required to meaningfully inhibit the serotonin transporter (SERT), a pharmacological threshold necessary to achieve clinically relevant antidepressant effects. At these levels, trazodone acts as a multimodal serotonergic agent, combining SERT inhibition with continued antagonism at 5-HT_2A_ and 5-HT_2C_ receptors, which classifies it within the serotonin antagonist and reuptake inhibitors: SARIs category (Stahl, 2009; Fagiolini et al., 2023). This pharmacological profile, combined with partial 5-HT_1A_ receptor agonism, may underlie an enhanced anxiolytic mechanism of action, operating not only through complex serotonergic modulation but also via the engagement of additional neurotransmitter systems and downstream intracellular signaling pathways (Celada et al., 2004; Odagaki et al., 2005; Stahl, 2009; Fagiolini et al., 2023; Lin et al., 2023). Beyond the aforementioned effects, trazodone acts as an antagonist at 5-HT_7_ and α_2_ receptors, exhibits negligible activity at muscarinic, dopaminergic, and GABAergic receptors, and, although it binds to histaminergic targets, the functional significance of this binding has yet to be fully elucidated (Albert et al., 2021; Oggianu et al., 2022). Treatment with trazodone may be associated with somnolence, headache, and dry mouth, whereas orthostatic hypotension occurs less frequently, likely attributable to peripheral α_1_-adrenergic receptor antagonism (Fagiolini et al., 2023). It should be noted that the potential severity of these effects depends on the formulation and the pharmacokinetic profile of the drug. The immediate-release (IR) formulation is associated with a rapid onset and high peak plasma levels, which in turn increase the likelihood of undesirable effects. In contrast, the extended-release (XR; termed OAD: Once-A-Day) reformulation of trazodone produces a slower rise in plasma concentrations, with a delayed peak and a gradual reduction, which may contribute to improved tolerability (Fagiolini et al., 2020).

As part of the TED project, the effectiveness of trazodone XR and SSRIs was compared over a 12-week naturalistic, experiment in patients with MDD (Dudek et al., 2023; Siwek et al., 2023; Siwek et al., 2024; Siwek et al., 2025). Using the mixed model for repeated measures, the pilot study demonstrated the superior efficacy of trazodone XR over SSRIs, primarily in reducing depressive symptoms, as assessed by the Quick Inventory of Depressive Symptomatology–clinician rated (QIDS-CR), and in alleviating insomnia severity, as measured by the Athens Insomnia Scale (AIS) (Siwek et al., 2023). In a subsequent study, using the same statistical model, trazodone XR demonstrated greater efficacy in reducing depressive symptoms, as measured by the Montgomery–Åsberg Depression Rating Scale (MADRS) and the self-rated version of the QIDS (QIDS-SR), as well as anxiety symptoms assessed with the Hamilton Anxiety Rating scale (HAM-A) and insomnia measured by the AIS, compared to SSRIs (Dudek et al., 2023). Furthermore, in another study it showed greater improvements than SSRIs in certain domains of health-related quality of life (HRQoL) (Siwek et al., 2024). Finally, a study included patients treated with trazodone XR as a first choice and those who received it after unsuccessful treatment with SSRIs, and it demonstrated that in the latter group treatment efficacy was comparable to that observed in patients for whom trazodone XR was used as the first-line therapy (Siwek et al., 2025).

The present study builds upon previous analyses conducted within this project and assesses the effects of the antidepressant drugs under investigation using effect sizes as the primary measure. This metric indicates how substantial a difference or effect is, offering insight into its real-world relevance—not just whether it exists. In contrast, p-values only indicate the probability that results are due to chance, without reflecting their magnitude. Especially in large-scale trials, negligible effects may reach statistical significance while lacking clinical importance (Ranganathan et al., 2015). Effect size provides a more precise basis for interpretation and facilitates comparisons across studies, thereby enabling the detection of beneficial treatments even in trials with a small number of patients (McGough and Faraone, 2009). Therefore, reporting both p-values and effect sizes is recommended to achieve a thorough understanding of research outcomes (McGough and Faraone, 2009; Sullivan and Feinn, 2012; Ranganathan et al., 2015; Schober et al., 2018).

### 1.1 Aim of the study

This study investigates the magnitude and trajectory of clinical improvement, expressed as effect sizes, associated with trazodone and SSRIs across multiple time points. Within-group changes in depressive symptomatology from baseline to subsequent weeks were analyzed separately for each treatment, followed by between-group comparisons to assess differences in the rate and extent of symptom reduction over time. To capture a comprehensive picture of treatment effects, multiple validated clinical scales were used, each targeting distinct symptom domains. These instruments assessed various dimensions of MDD and related symptoms, including depressed mood, anhedonia, anxiety, sleep disturbances in real-world settings, enabling a nuanced evaluation of treatment efficacy.

## 2 Materials and methods

### 2.1 Study design

The present study employed the same methodological design as previously used in the pilot and follow-up studies within this research series, allowing for consistent analysis throughout the successive stages of the project (Dudek et al., 2023; Siwek et al., 2023; Siwek et al., 2024; Siwek et al., 2025). A brief overview of the methodology is provided below; for full details, see: (Dudek et al., 2023).

A single-center, non-randomized, open-label study employing a non-inferiority design within a naturalistic observational framework was carried out to evaluate and compare the therapeutic efficacy and tolerability profiles of trazodone XR and SSRIs (used as single-agent treatments: citalopram, escitalopram, paroxetine or sertraline), administered over a period of 12 weeks (Dudek et al., 2023; Siwek et al., 2023; Siwek et al., 2024; Siwek et al., 2025). In this study, SSRIs and trazodone XR were dose-adjusted in accordance with label-approved therapeutic dosing, reflecting real-world clinical practice, with dose selection and titration performed at the treating clinician’s discretion within standard ranges. Individuals aged 18–65 years with a first-onset episode of MDD or a current relapse in the course of recurrent depression, diagnosed in accordance with the criteria of the Diagnostic and Statistical Manual of Mental Disorders, Fifth Edition (DSM-5), were eligible for inclusion (Dudek et al., 2023). This study was performed following the principles of the Declaration of Helsinki, with authorization from the Bioethics Committee of the Jagiellonian University in Krakow, Poland (approval no. 1072.6120.113.2021). Written informed consent was obtained from all participants prior to study enrollment.

### 2.2 Clinical assessment

Evaluations were performed at five predefined time points—baseline (week 0), week 2, week 4, week 8, and week 12 of the treatment period—following the procedures outlined in previous publications (Dudek et al., 2023; Siwek et al., 2023; Siwek et al., 2024; Siwek et al., 2025). To assess the severity of core psychopathological symptoms, a set of validated clinician- and self-rated instruments was utilized. The study’s primary outcome measure, depressive symptomatology, was evaluated using multiple instruments, with the QIDS (16-item) demonstrating particular utility owing to its dual-format design, which includes both self-rated and clinician-rated assessments. This enabled a more comprehensive assessment of symptom severity by incorporating patient perspectives (Rush et al., 2003). The QIDS also captures the frequency and intensity of core depressive features, facilitating standardized evaluation of treatment-related changes over time (Dudek et al., 2023; Siwek et al., 2025). Furthermore, the MADRS was utilized to assess the severity of depressive symptoms (Montgomery and Asberg, 1979; Iannuzzo et al., 2006). This clinician-administered instrument comprises 10 items covering core dimensions of depression and is widely used in clinical trials to monitor treatment efficacy and symptom progression (Montgomery and Asberg, 1979).

Secondary endpoints included anhedonia, evaluated using the Snaith–Hamilton Pleasure Scale (SHAPS), a self-report instrument assessing the ability to experience pleasure in daily life; anxiety, measured with the clinician-administered HAM-A, which captures both psychic and somatic symptoms of anxiety; and sleep disturbances, assessed via the AIS, a self-report tool evaluating sleep quality and insomnia symptoms (Hamilton, 1959; Maier et al., 1988; Snaith et al., 1995; Soldatos et al., 2003; Mateen et al., 2017; Trøstheim et al., 2020).

### 2.3 Statistical assessment

Statistical analysis followed the approach adopted in our earlier research, where an extensive description of the methodology can be found (Dudek et al., 2023; Siwek et al., 2024; Siwek et al., 2025). Briefly, all statistical calculations were carried out using data from 79 patients treated with trazodone and 81 patients treated with SSRIs. Baseline group differences in demographic and clinical characteristics were examined using independent-samples t-tests for continuous variables (reported as mean ± SD) and chi-square (χ^2^) tests for categorical data (presented as proportions). The Shapiro–Wilk test was used to assess the normality of continuous variables (Dudek et al., 2023).

Effect sizes (Cohen’s d) were determined to quantify changes in depressive symptomatology, as assessed by the QIDS-SR, QIDS-CR, MADRS, SHAPS, HAM-A, and AIS scales, from baseline (week 0) through follow-up evaluations conducted at weeks 2, 4, 8, and 12. Interpretation of effect sizes followed conventional thresholds, with values of 0.2, 0.5, and 0.8 considered indicative of small, moderate, and large effects, respectively (Cohen, 1988; Lakens, 2013; Maher et al., 2013). Paired-sample t-tests were conducted to evaluate within-group changes across time points for each treatment arm. Statistical significance was set at p < 0.05 (two-tailed), and results were reported to indicate the magnitude and consistency of symptom change over the course of treatment. All statistical analyses were performed using R statistical software: version 4.5.1 (R Core Team, 2025) R: A Language and Environment for Statistical Computing. R Foundation for Statistical Computing).

## 3 Results

Comprehensive baseline characteristics have been reported in detail in a previous publication; in the present analysis, only the most salient between-group differences are briefly discussed (Dudek et al., 2023). At baseline, both treatment groups were broadly similar across most demographic and clinical variables. However, the trazodone XR group showed significantly higher rates of smoking (p < 0.02) and a longer history of psychiatric treatment (p < 0.05) compared to patients receiving SSRIs (Dudek et al., 2023).

### 3.1 Primary endpoints

#### 3.1.1 QIDS-SR patient-rated outcome

**FIGURE 1:**
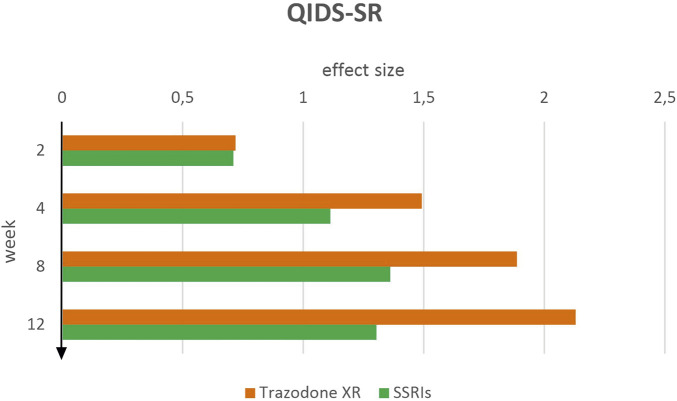
QIDS-CR effect sizes over time for trazodone XR and SSRIs.

Effect sizes for QIDS-SR indicated a consistently greater reduction in depressive symptoms in the trazodone XR group compared to SSRIs across all assessed time points (Figure 1). Following 2 weeks of therapy, trazodone XR showed a moderate effect size of *d* = 0.72 (95% CI [0.36–1.08]), which was comparable to the SSRI group at *d* = 0.71 (95% CI [0.38–1.04]). The effect size, observed at week 4 for trazodone XR increased markedly to *d* = 1.49 (95% CI [1.13–1.86]), exceeding that of SSRIs at *d* = 1.11 (95% CI [0.78–1.44]). This trend continued at week 8, where trazodone XR reached *d* = 1.89 (95% CI [1.51–2.26]), compared to *d* = 1.36 (95% CI [1.03–1.70]) for SSRIs. At week 12, trazodone XR demonstrated the largest effect size observed in the study (*d* = 2.13, 95% CI [1.75–2.51]), while the SSRI group plateaued at *d* = 1.30 (95% CI [0.96–1.64]).

The results point to a potentially earlier and progressively greater therapeutic effect of trazodone XR from the patient’s perspective.

Furthermore paired-sample t-tests showed that both trazodone XR and SSRIs were associated with significant reductions in symptom severity from baseline to weeks 2, 4, 8, and 12, as well as from week 2 to subsequent time points (Table 1). For trazodone XR, a significant contrast was also observed between weeks 4 and 12, whereas for SSRIs, comparisons between later time points (weeks 4 vs. 8, 4 vs. 12, and 8 vs. 12) were non-significant, suggesting a plateau effect after the initial improvements.

**TABLE 1:** Results of t-tests for changes in QIDS-SR scores during treatment with trazodone XR (T-XR) and SSRIs across time points.

T-XR baseline (week 0)	Mean start (SD)	Mean end (SD)	t (df)	p-value
vs. week 2	15.74 (0.68)	12.03 (0.63)	6.04 (51)	<0.0001 ****
vs. week 4		8.05 (0.64)	9.44 (50)	<0.0001 ****
vs. week 8		6.02 (0.66)	10.8 (47)	<0.0001 ****
vs. week 12		4.76 (0.68)	10.1 (44)	<0.0001 ****

SSRIs baseline (week 0)	Mean start (SD)	Mean end (SD)	t (df)	p-value
vs. week 2	14.45 (0.60)	10.79 (0.60)	6.34 (65)	<0.0001 ****
vs. week 4		8.72 (0.60)	8.61 (67)	<0.0001 ****
vs. week 8		7.44 (0.61)	9.91 (64)	<0.0001 ****
vs. week 12		7.73 (0.63)	7.80 (59)	<0.0001 ****

T-XR (week 2)	Mean start (SD)	Mean end (SD)	t (df)	p-value
vs. week 4	12.03 (0.63)	8.05 (0.64)	6.50 (58)	<0.0001 ****
vs. week 8		6.02 (0.66)	6.88 (54)	<0.0001 ****
vs. week 12		4.76 (0.68)	7.34 (51)	<0.0001 ****

SSRIs (week 2)	Mean start (SD)	Mean end (SD)	t (df)	p-value
vs. week 4	10.79 (0.60)	8.72 (0.60)	4.45 (70)	<0.001 ***
vs. week 8		7.44 (0.61)	5.38 (67)	<0.0001 ****
vs. week 12		7.73 (0.63)	4.04 (63)	<0.01 **

T-XR (week 4)	Mean start (SD)	Mean end (SD)	t (df)	p-value
vs. week 8	8.05 (0.64)	6.02 (0.66)	2.43 (55)	0.182 ns
vs. week 12		4.76 (0.68)	3.80 (50)	<0.01 **

SSRIs (week 4)	Mean start (SD)	Mean end (SD)	t (df)	p-value
vs. week 8	8.72 (0.60)	7.44 (0.61)	2.48 (69)	0.157 ns
vs. week 12		7.73 (0.63)	1.52 (65)	1.00 ns

T-XR (week 8)	Mean start (SD)	Mean end (SD)	t (df)	p-value
vs. week 12	6.02 (0.66)	4.76 (0.68)	1.54 (54)	1.00 ns

SSRIs (week 8)	Mean start (SD)	Mean end (SD)	t (df)	p-value
vs. week 12	7.44 (0.61)	7.73 (0.63)	−0.29 (63)	1.00 ns

#### 3.1.2 QIDS-CR clinician-rated outcome

**FIGURE 2:**
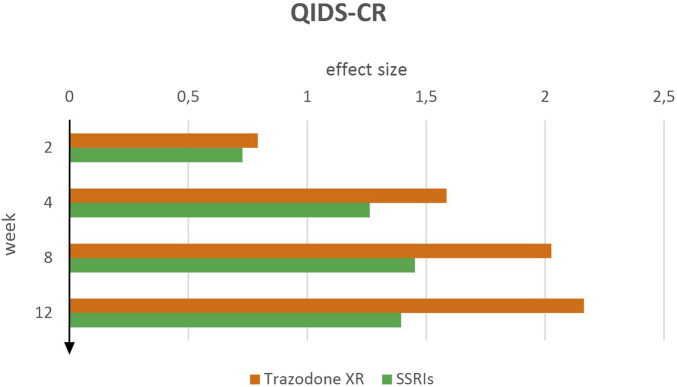
QIDS-CR effect sizes over time for trazodone XR and SSRIs.

Effect sizes for QIDS-CR showed greater symptom improvement in the trazodone XR group compared to SSRIs across all time points (Figure 2). By week 2 of treatment, both groups showed similar effects: trazodone XR demonstrated an effect size of *d* = 0.79 (95% CI [0.46–1.12]), while SSRIs achieved *d* = 0.73 (95% CI [0.40–1.05]). Within 4 weeks, both treatments achieved large effect sizes; however, trazodone XR showed a stronger effect (d = 1.59, 95% CI [1.25–1.93]) compared with SSRIs (d = 1.26, 95% CI [0.94–1.59]). This pattern continued at week 8, where trazodone XR showed a large and increasing effect of *d* = 2.03 (95% CI [1.67–2.38]), compared to *d* = 1.45 (95% CI [1.12–1.79]) for SSRIs. At week 12, trazodone XR maintained the strongest treatment effect observed in the study, with *d* = 2.16 (95% CI [1.80–2.52]), while the SSRI group plateaued at *d* = 1.39 (95% CI [1.06–1.73]). Between-group differences consistently favored trazodone XR across all time points, becoming especially marked from week 4 onward.

According to standard thresholds, these findings suggest a consistently stronger clinician-assessed therapeutic effect of trazodone XR over time. This effect appears to be not only more pronounced but also earlier in onset compared to SSRIs, with between-group differences emerging from week 4 and gradually increasing throughout the follow-up period.

In the paired-sample *t*-tests indicated both trazodone XR and SSRIs were associated with significant reductions in QIDS-CR scores from baseline to weeks 2, 4, 8, and 12 (Table 2). For trazodone XR, additional improvements were also observed when comparing week 2 with weeks 4, 8, and 12, and between weeks 4 with 8 and 12. In the SSRI group, significant decreases were seen also from week 2 to later time points; however, contrasts between later assessments (weeks 4 vs. 8, 4 vs. 12, 8 vs. 12) were not significant, suggesting that symptom reduction plateaued after the initial treatment phase.

**TABLE 2:** Results of t-tests for changes in QIDS-CR scores during treatment with trazodone XR (T-XR) and SSRIs across time points.

T-XR baseline (week 0)	Mean start (SD)	Mean end (SD)	t (df)	p-value
vs. week 2	14.21 (0.60)	10.15 (0.61)	7.28 (65)	<0.0001 ****
vs. week 4		6.09 (0.61)	12.3 (64)	<0.0001 ****
vs. week 8		3.83 (0.64)	14.9 (60)	<0.0001 ****
vs. week 12		3.13 (0.66)	12.9 (58)	<0.0001 ****

SSRIs baseline (week 0)	Mean start (SD)	Mean end (SD)	t (df)	p-value
vs. week 2	13.77 (0.58)	10.04 (0.60)	7.13 (67)	<0.0001 ****
vs. week 4		7.30 (0.60)	9.86 (67)	<0.0001 ****
vs. week 8		6.33 (0.63)	8.90 (62)	<0.0001 ****
vs. week 12		6.63 (0.63)	8.66 (61)	<0.0001 ****

T-XR (week 2)	Mean start (SD)	Mean end (SD)	t (df)	p-value
vs. week 4	10.15 (0.61)	6.09 (0.61)	6.76 (65)	<0.0001 ****
vs. week 8		3.83 (0.64)	8.56 (61)	<0.0001 ****
vs. week 12		3.13 (0.66)	7.26 (57)	<0.0001 ****

SSRIs (week 2)	Mean start (SD)	Mean end (SD)	t (df)	p-value
vs. week 4	10.04 (0.60)	7.30 (0.60)	5.31 (66)	<0.0001 ****
vs. week 8		6.33 (0.63)	4.87 (63)	<0.0001 ****
vs. week 12		6.63 (0.63)	3.96 (60)	<0.01 **

T-XR (week 4)	Mean start (SD)	Mean end (SD)	t (df)	p-value
vs. week 8	6.09 (0.61)	3.83 (0.64)	3.58 (62)	<0.01 **
vs. week 12		3.13 (0.66)	3.10 (58)	<0.05 *

SSRIs (week 4)	Mean start (SD)	Mean end (SD)	t (df)	p-value
vs. week 8	7.30 (0.60)	6.33 (0.63)	1.95 (63)	0.558 ns
vs. week 12		6.63 (0.63)	0.47 (62)	1.00 ns

T-XR (week 8)	Mean start (SD)	Mean end (SD)	t (df)	p-value
vs. week 12	3.83 (0.64)	3.13 (0.66)	0.64 (59)	1.00 ns

SSRIs (week 8)	Mean start (SD)	Mean end (SD)	t (df)	p-value
vs. week 12	6.33 (0.63)	6.63 (0.63)	−0.19 (59)	1.00 ns

#### 3.1.3 MADRS clinician-rated depression severity

**FIGURE 3:**
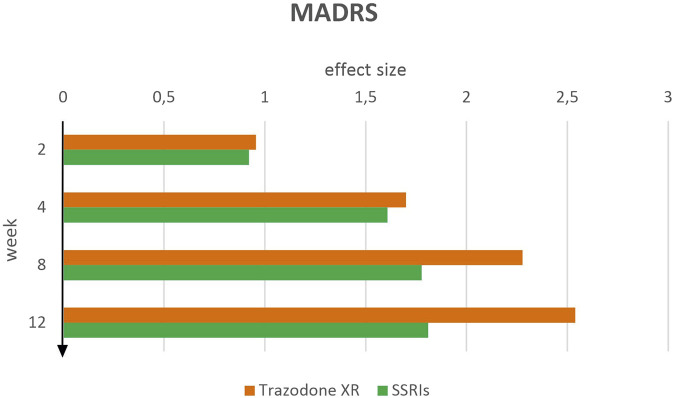
MADRS effect sizes over time for trazodone XR and SSRIs.

The therapeutic effect of trazodone XR exceeded that observed in the SSRIs group across all time points (Figure 3). However, at the initial 2-week assessment, trazodone XR achieved an effect size of d = 0.96 (95% CI [0.62–1.29]), while SSRIs showed a similar improvement of d = 0.92 (95% CI [0.60–1.25]). Likewise, at week 4, effect sizes were comparable for both treatments: trazodone XR increased to d = 1.70 (95% CI [1.35–2.05]), whereas SSRIs reached d = 1.61 (95% CI [1.28–1.94]). At week 8, trazodone XR demonstrated a substantially greater effect of d = 2.28 (95% CI [1.92–2.64]) compared to d = 1.78 (95% CI [1.43–2.12]) for SSRIs, and by week 12, trazodone XR maintained the highest treatment effect observed in the study, with d = 2.54 (95% CI [2.17–2.91]), while the SSRI group plateaued at d = 1.81 (95% CI [1.47–2.15]). Between-group differences favored trazodone XR from week 8 onward, becoming more pronounced thereafter and indicating a stronger and earlier therapeutic effect compared to SSRIs.

In line with the scales described above, paired-sample t-tests demonstrated significant reductions in MADRS scores for both trazodone XR and SSRIs from baseline to weeks 2, 4, 8, and 12, as well as from week 2 to later time points (Table 3). For trazodone XR, additional improvements were also observed between week 4 and weeks 8 and 12, whereas in the SSRI group, all later contrasts (weeks 4 vs. 8, 4 vs. 12, and 8 vs. 12) were not statistically significant.

**TABLE 3:** Results of t-tests for changes in MADRS scores during treatment with trazodone XR (T-XR) and SSRIs across time points.

T-XR baseline (week 0)	Mean start (SD)	Mean end (SD)	t (df)	p-value
vs. week 2	28.76 (1.05)	20.23 (1.07)	8.59 (64)	<0.0001 ****
vs. week 4		13.59 (1.10)	12.7 (60)	<0.0001 ****
vs. week 8		8.43 (1.12)	17.0 (58)	<0.0001 ****
vs. week 12		6.10 (1.15)	16.4 (57)	<0.0001 ****

SSRIs baseline (week 0)	Mean start (SD)	Mean end (SD)	t (df)	p-value
vs. week 2	27.25 (1.02)	19.03 (1.04)	9.44 (68)	<0.0001 ****
vs. week 4		12.89 (1.05)	13.6 (66)	<0.0001 ****
vs. week 8		11.38 (1.10)	12.1 (61)	<0.0001 ****
vs. week 12		11.09 (1.11)	11.7 (60)	<0.0001 ****

T-XR (week 2)	Mean start (SD)	Mean end (SD)	t (df)	p-value
vs. week 4	20.23 (1.07)	13.59 (1.10)	6.03 (59)	<0.0001 ****
vs. week 8		8.43 (1.12)	9.89 (59)	<0.0001 ****
vs. week 12		6.10 (1.15)	8.78 (56)	<0.0001 ****

SSRIs (week 2)	Mean start (SD)	Mean end (SD)	t (df)	p-value
vs. week 4	19.03 (1.04)	12.89 (1.05)	6.75 (66)	<0.0001 ****
vs. week 8		11.38 (1.10)	6.11 (60)	<0.0001 ****
vs. week 12		11.09 (1.11)	6.13 (59)	<0.0001 ****

T-XR (week 4)	Mean start (SD)	Mean end (SD)	t (df)	p-value
vs. week 8	13.59 (1.10)	8.43 (1.12)	4.37 (56)	<0.0001 ****
vs. week 12		6.10 (1.15)	5.58 (53)	<0.001 ***

SSRIs (week 4)	Mean start (SD)	Mean end (SD)	t (df)	p-value
vs. week 8	12.89 (1.05)	11.38 (1.10)	1.11 (61)	0.27 ns
vs. week 12		11.09 (1.11)	1.17 (59)	0.25 ns

T-XR (week 8)	Mean start (SD)	Mean end (SD)	t (df)	p-value
vs. week 12	8.43 (1.12)	6.10 (1.15)	1.27 (56)	0.21 ns

SSRIs (week 8)	Mean start (SD)	Mean end (SD)	t (df)	p-value
vs. week 12	11.38 (1.10)	11.09 (1.11)	−0.458 (57)	0.65 ns

### 3.2 Secondary endpoints

#### 3.2.1 SHAPS anhedonia–patient-reported outcome

**FIGURE 4:**
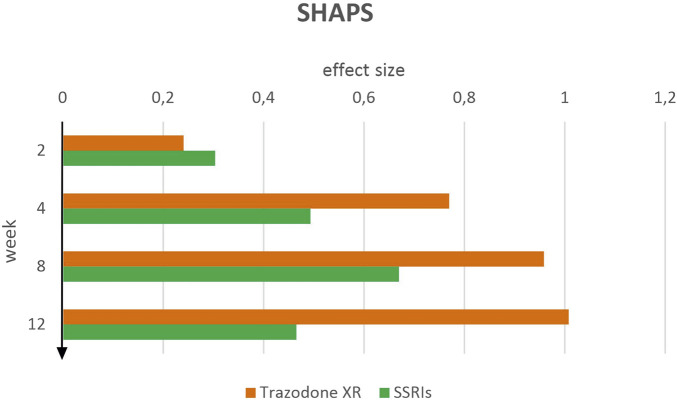
SHAPS effect sizes over time for trazodone XR and SSRIs.

Greater improvement in anhedonia symptoms was observed in the trazodone XR group compared to SSRIs at most time points, as reflected by effect sizes on the SHAPS scale (Figure 4). At week 2 of therapy, both treatments showed small effects, with trazodone XR yielding d = 0.24 (95% CI [–0.08–0.57]) and SSRIs d = 0.30 (95% CI [–0.01–0.62]). At week 4, the effect size for trazodone XR increased to d = 0.77 (95% CI [0.43–1.11]), representing a moderate effect, whereas SSRIs showed a smaller improvement with d = 0.49 (95% CI [0.17–0.84]), indicating a small-to-moderate effect. At week 8, trazodone XR demonstrated a large effect size of d = 0.96 (95% CI [0.62–1.30]), while SSRIs remained within the moderate range with d = 0.67 (95% CI [0.35–1.01]). By week 12, the difference between treatments became more pronounced, with trazodone XR reaching a large effect size of d = 1.01 (95% CI [0.66–1.35]), in contrast to SSRIs, which maintained a small-to-moderate effect size of d = 0.47 (95% CI [0.14–0.79]).

These findings suggest a stronger and steadily increasing impact of trazodone XR on improving anhedonia compared to SSRIs, with clearer between-group differences emerging from week 4 and becoming more evident over time.

Significant reductions in SHAPS scores from baseline to weeks 4, 8 and 12 were observed for trazodone XR, but not from baseline to week 2 (Table 4). Additional reductions were also significant from week 2 to weeks 4, 8 and 12, but not between later time points (weeks 4 vs. 8, 4 vs. 12 and 8 vs. 12). For SSRIs, significant reductions were observed from baseline to weeks 2, 4, 8 and 12, whereas comparisons from week 2 to weeks 4, 8 and 12 and between later time points (weeks 4 vs. 8, 4 vs. 12 and 8 vs. 12) were not statistically significant.

**TABLE 4:** Results of t-tests for changes in SHAPS scores during treatment with trazodone XR (T-XR) and SSRIs across time points.

T-XR baseline (week 0)	Mean start (SD)	Mean end (SD)	t (df)	p-value
vs. week 2	6.69 (0.50)	5.67 (0.49)	2.10 (69)	0.039 ns
vs. week 4		3.41 (0.53)	4.95 (62)	<0.0001 ****
vs. week 8		2.60 (0.54)	5.94 (59)	<0.0001 ****
vs. week 12		2.39 (0.55)	6.08 (58)	<0.0001 ****

SSRIs baseline (week 0)	Mean start (SD)	Mean end (SD)	t (df)	p-value
vs. week 2	6.64 (0.47)	5.35 (0.49)	4.10 (73)	<0.01 **
vs. week 4		4.54 (0.51)	5.81 (70)	<0.0001 ****
vs. week 8		3.78 (0.50)	5.26 (72)	<0.0001 ****
vs. week 12		4.66 (0.52)	4.17 (66)	<0.001 ***

T-XR (week 2)	Mean start (SD)	Mean end (SD)	t (df)	p-value
vs. week 4	5.67 (0.49)	3.41 (0.53)	4.11 (65)	<0.01 **
vs. week 8		2.60 (0.54)	4.46 (62)	<0.001 ***
vs. week 12		2.39 (0.55)	3.88 (60)	<0.01 **

SSRIs (week 2)	Mean start (SD)	Mean end (SD)	t (df)	p-value
vs. week 4	5.35 (0.49)	4.54 (0.51)	2.50 (69)	0.015 ns
vs. week 8		3.78 (0.50)	2.81 (71)	0.006 ns
vs. week 12		4.66 (0.52)	1.42 (64)	0.161 ns

T-XR (week 4)	Mean start (SD)	Mean end (SD)	t (df)	p-value
vs. week 8	3.41 (0.53)	2.60 (0.54)	0.99 (56)	0.326 ns
vs. week 12		2.39 (0.55)	0.98 (54)	0.331 ns

SSRIs (week 4)	Mean start (SD)	Mean end (SD)	t (df)	p-value
vs. week 8	4.54 (0.51)	3.78 (0.50)	1.33 (68)	0.189 ns
vs. week 12		4.66 (0.52)	0.02 (62)	0.983 ns

T-XR (week 8)	Mean start (SD)	Mean end (SD)	t (df)	p-value
vs. week 12	2.60 (0.54)	2.39 (0.55)	0.13 (59)	0.901 ns

SSRIs week 8	Mean start (SD)	Mean end (SD)	t (df)	p-value
vs. week 12	3.78 (0.50)	4.66 (0.52)	−1.68 (66)	0.098 ns

#### 3.2.2 HAM-A clinician-rated anxiety severity

**FIGURE 5:**
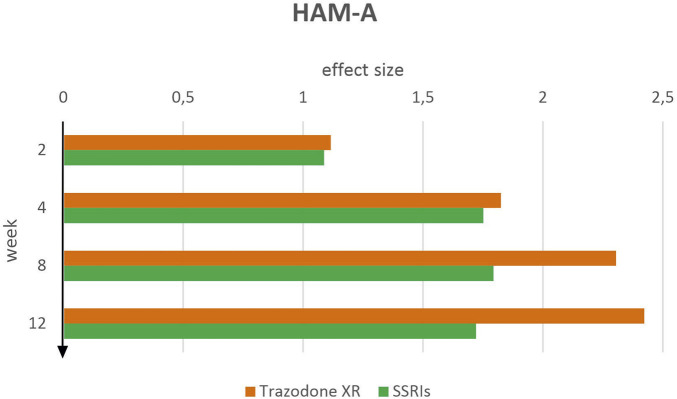
HAM-A effect sizes over time for trazodone XR and SSRIs.

Effect sizes for HAM-A indicated greater anxiety symptom improvement in the trazodone XR group compared to SSRIs across all measured time points (Figure 5). At week 2, both treatment groups demonstrated large effects: trazodone XR (d = 1.12, 95% CI [0.78–1.46]) and SSRIs (d = 1.09, 95% CI [0.77–1.41]). At week 4, the effect sizes remained large for both groups, with a slight advantage for trazodone XR (d = 1.82, 95% CI [1.48–2.18]) over SSRIs (d = 1.75, 95% CI [1.42–2.08]). Trazodone XR demonstrated a notably stronger effect (d = 2.30, 95% CI [1.94–2.67]) compared to SSRIs (d = 1.79, 95% CI [1.46–2.13]), at week 8 of treatment. At week 12, trazodone XR showed the highest effect size observed across the entire study period (d = 2.42, 95% CI [2.05–2.79]), exceeding the effect for SSRIs (d = 1.72, 95% CI [1.38–2.06]).

These results suggest a consistently greater and progressively increasing anxiolytic effect of trazodone XR over time compared to SSRIs. While both treatments produced large effects early in the course of therapy, the difference between groups became more pronounced from week 8 onward, favoring trazodone XR as a more effective intervention for anxiety symptoms.

Significant reductions in HAM-A scores from baseline and from weeks 2 and 4 to subsequent time points up to week 12 were observed for trazodone XR as determined by paired-sample t-tests (Table 5). However, comparisons between weeks 8 and 12 were not statistically significant. For SSRIs, reductions were significant from baseline to week 2, 4, 8 and 12 and from week 2 to week 4, 8 and 12, but not between later time points (weeks 4 vs. 8, 4 vs. 12 and 8 vs. 12).

**TABLE 5:** Results of t-tests for changes in HAM-A scores during treatment with trazodone XR (T-XR) and SSRIs across time points.

T-XR baseline (week 0)	Mean start (SD)	Mean end (SD)	t (df)	p-value
vs. week 2	21.49 (0.91)	13.23 (0.89)	11.4 (60)	<0.0001 ****
vs. week 4		7.97 (0.89)	14.7 (61)	<0.0001 ****
vs. week 8		4.42 (0.92)	20.3 (59)	<0.0001 ****
vs. week 12		3.54 (0.95)	16.9 (57)	<0.0001 ****

SSRIs baseline (week 0)	Mean start (SD)	Mean end (SD)	t (df)	p-value
vs. week 2	20.58 (0.84)	12.52 (0.86)	10.5 (70)	<0.0001 ****
vs. week 4		7.60 (0.87)	12.5 (68)	<0.0001 ****
vs. week 8		7.29 (0.87)	12.4 (69)	<0.0001 ****
vs. week 12		7.83 (0.89)	11.2 (65)	<0.0001 ****

T-XR (week 2)	Mean start (SD)	Mean end (SD)	t (df)	p-value
vs. week 4	13.23 (0.89)	7.97 (0.89)	6.76 (64)	<0.0001 ****
vs. week 8		4.42 (0.92)	9.71 (60)	<0.0001 ****
vs. week 12		3.54 (0.95)	7.95 (57)	<0.0001 ****

SSRIs (week 2)	Mean start (SD)	Mean end (SD)	t (df)	p-value
vs. week 4	12.52 (0.86)	7.60 (0.87)	6.10 (69)	<0.0001 ****
vs. week 8		7.29 (0.87)	5.38 (70)	<0.0001 ****
vs. week 12		7.83 (0.89)	4.25 (66)	<0.001 ***

T-XR (week 4)	Mean start (SD)	Mean end (SD)	t (df)	p-value
vs. week 8	7.97 (0.89)	4.42 (0.92)	4.98 (61)	<0.0001 ****
vs. week 12		3.54 (0.95)	3.76 (58)	<0.01 **

SSRIs (week 4)	Mean start (SD)	Mean end (SD)	t (df)	p-value
vs. week 8	7.60 (0.87)	7.29 (0.87)	0.68 (68)	0.499 ns
vs. week 12		7.83 (0.89)	0.09 (65)	0.926 ns

T-XR (week 8)	Mean start (SD)	Mean end (SD)	t (df)	p-value
vs. week 12	4.42 (0.92)	3.54 (0.95)	0.17 (59)	0.863 ns

SSRIs (week 8)	Mean start (SD)	Mean end (SD)	t (df)	p-value
vs. week 12	7.29 (0.87)	7.83 (0.89)	−0.87 (67)	0.387 ns

#### 3.2.3 AIS patient-reported insomnia severity

**FIGURE 6:**
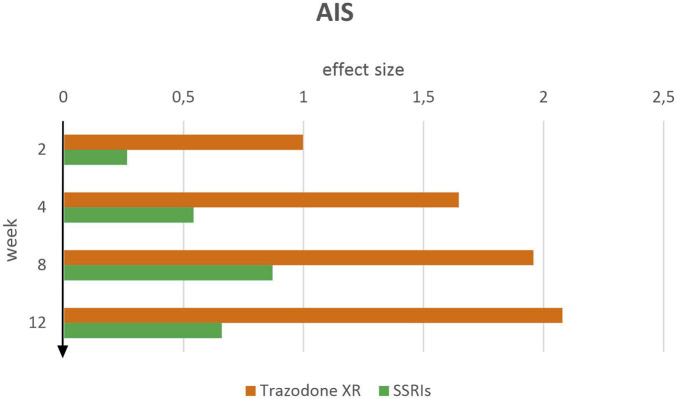
AIS effect sizes over time for trazodone XR and SSRIs.

At week 2, trazodone XR showed a large effect size in the AIS (*d* = 1.00, 95% CI [0.67–1.32]), while SSRIs demonstrated a small (*d* = 0.27, 95% CI [–0.05–0.58]) (Figure 6). Trazodone XR achieved a substantially greater effect size (*d* = 1.65, 95% CI [1.30–1.99]) than SSRIs (*d* = 0.54, 95% CI [0.22–0.86]) by week 4 of the experiment. This advantage persisted at week 8, with trazodone XR reaching *d* = 1.96 (95% CI [1.61–2.31]) compared to *d* = 0.87 (95% CI [0.55–1.19]) for SSRIs. By week 12, trazodone XR maintained the highest effect observed in the study (*d* = 2.08, 95% CI [1.72–2.44]), while the SSRI group demonstrated only a moderate improvement (*d* = 0.66, 95% CI [0.33–0.99]).

These findings indicate a consistently stronger impact of trazodone XR on insomnia symptoms over time, with clear superiority over SSRIs evident from the earliest measurement point and becoming increasingly pronounced through week 12.

Trazodone XR and SSRIs were associated with significant reductions in AIS scores from baseline, as well as from week 2 to subsequent weeks of treatment, as determined by paired-sample t-tests (Table 6). For trazodone XR, comparisons between week 4 and later time points (weeks 8 and 12) were not statistically significant. For SSRIs, a significant reduction was observed between week 4 vs. 8, but not between week 4 vs. 12. Furthermore, comparisons between weeks 8 and 12 were not statistically significant for either treatment.

**TABLE 6:** Results of t-tests for changes in AIS scores during treatment with trazodone XR (T-XR) and SSRIs across time points.

T-XR baseline (week 0)	Mean start (SD)	Mean end (SD)	t (df)	p-value
vs. week 2	13.73 (0.56)	8.96 (0.55)	7.97 (70)	<0.0001 ****
vs. week 4		5.85 (0.58)	10.3 (63)	<0.0001 ****
vs. week 8		4.37 (0.60)	10.9 (61)	<0.0001 ****
vs. week 12		3.79 (0.61)	9.70 (60)	<0.0001 ****

SSRIs baseline (week 0)	Mean start (SD)	Mean end (SD)	t (df)	p-value
vs. week 2	10.38 (0.54)	9.11 (0.56)	3.66 (69)	<0.01 **
vs. week 4		7.79 (0.55)	5.59 (72)	<0.0001 ****
vs. week 8		6.22 (0.56)	7.52 (70)	<0.0001 ****
vs. week 12		7.22 (0.58)	6.60 (64)	<0.0001 ****

T-XR (week 2)	Mean start (SD)	Mean end (SD)	t (df)	p-value
vs. week 4	8.96 (0.55)	5.85 (0.58)	6.46 (66)	<0.0001 ****
vs. 8 weeks		4.37 (0.60)	6.22 (62)	<0.0001 ****
vs. week 12		3.79 (0.61)	5.51 (60)	<0.0001 ****

SSRIs (week 2)	Mean start (SD)	Mean end (SD)	t (df)	p-value
vs. week 4	9.11 (0.56)	7.79 (0.55)	3.13 (70)	<0.05 *
vs. week 8		6.22 (0.56)	5.34 (69)	<0.0001 ****
vs. week 12		7.22 (0.58)	3.08 (62)	<0.05 *

T-XR (week 4)	Mean start (SD)	Mean end (SD)	t (df)	p-value
vs. week 8	5.85 (0.58)	4.37 (0.60)	1.89 (59)	0.63 ns
vs. week 12		3.79 (0.61)	2.02 (57)	0.48 ns

SSRIs (week 4)	Mean start (SD)	Mean end (SD)	t (df)	p-value
vs. week 8	7.79 (0.55)	6.22 (0.56)	3.76 (72)	<0.01 **
vs. week 12		7.22 (0.58)	1.19 (66)	0.24 ns

T-XR (week 8)	Mean start (SD)	Mean end (SD)	t (df)	p-value
vs. week 12	4.37 (0.60)	3.79 (0.61)	0.666 (60)	1.0 ns

SSRIs (week 8)	Mean start (SD)	Mean end (SD)	t (df)	p-value
vs. week 12	6.22 (0.56)	7.22 (0.58)	−1.72 (66)	0.90 ns

## 4 Discussion

This comprehensive multi-faceted comparative analysis of effect sizes between trazodone XR and SSRIs, conducted across multiple scales including patient-reported measures, demonstrated previously unreported pronounced progressive increase in trazodone XR’s activity across successive time points.

In the depression severity scales QIDS-SR and QIDS-CR, a systematic improvement was observed at successive time points in the trazodone XR and SSRIs. Therapeutic effects were already evident at week 2, with greater improvement in the trazodone XR group compared to SSRIs. Over time, the magnitude of the effect increased, reaching its peak at week 8 for SSRIs and by week 12 for trazodone XR. Notably, SSRIs appeared to reach a plateau in clinical efficacy, while trazodone XR did not exhibit such a limitation. It consistently demonstrated greater efficacy than SSRIs in all assessments and at all time points, with differences becoming especially marked at the last two measurement points. It is worth emphasizing that a similar pattern was observed in both assessment methods, indicating that trazodone XR produced a more pronounced and sustained reduction in depressive symptoms over the 12-week period, regardless of whether severity was assessed subjectively or objectively, with the most marked differences emerging in the later stages of treatment. The progressive increase in the effect of trazodone XR observed until the end of treatment may reflect a cumulative benefit, encompassing both improvements in patients’ subjective perceptions and increasingly pronounced changes in clinician-rated assessments due to the drug’s sustained impact on mood. Notably, QIDS scales are often highly sensitive to improvements in somatic symptoms of depression, as patients perceive these changes as a meaningful enhancement of daily functioning (Brown et al., 2008). In our experiment, the favorable changes observed with trazodone XR may be attributable to its unique pharmacological and pharmacokinetic properties. As previously noted, trazodone is a multimodal antidepressant, and its effects arise not only from inhibition of the serotonin transporter but also from actions at serotonergic, histaminergic, and adrenergic receptors, which contribute to improving sleep quality, alleviating insomnia, and reducing the frequency of nocturnal awakenings (Stahl, 2009; Fagiolini et al., 2023).

Amelioration of sleep disturbances may potentiate the overall antidepressant effect and accelerate functional restoration, as reflected in these scales (Brown et al., 2008). In addition, the XR formulation of trazodone ensures more stable plasma concentrations, reducing fluctuations in drug activity, improving tolerability, and sustaining antidepressant effects, which may have contributed to the favorable progressive effect observed in these scales (Sheehan et al., 2009). Also in the MADRS scale, trazodone XR demonstrated a therapeutic advantage over SSRIs across all assessment points. While improvements were comparable between groups in the early phase of treatment, a marked increase in effect size for trazodone XR was observed from week 8 onward and sustained through the end of the study. In the QIDS-SR and QIDS-CR assessments, however, this superiority emerged earlier, around week 4, suggesting a more rapid onset of benefit in these measures. This initial separation is likely attributable to differences in the constructs and measurement sensitivity of these scales compared to the MADRS (Bernstein et al., 2010). The earlier superiority of trazodone XR in QIDS-SR and QIDS-CR may reflect, as previously noted, the scales’ greater sensitivity to early improvements in symptoms such as sleep and energy, which are rapidly influenced by trazodone’s multimodal mechanism (Rush et al., 2006).

Both trazodone XR and SSRIs demonstrated systematic improvement on the SHAPS scale, which assesses hedonic capacity across four domains: interests and leisure activities, social interaction, sensory experience, and food and drink, across successive assessment points (Snaith et al., 1995). Some effects were already apparent at week 2 of therapy, with SSRIs showing slightly higher effect size values at this early stage. However, from week 4 onward, trazodone XR consistently demonstrated greater clinical efficacy than SSRIs, with the difference between the groups becoming progressively more pronounced over time. In the trazodone XR group, the effect size increased steadily, reaching its maximum at week 12. In contrast, the SSRIs reached a lower peak at week 8, with values by week 12 falling back to levels similar to those observed by week 4. Presumably, this effect is mediated by trazodone’s antagonism of 5-HT_2A_ and 5-HT_2C_ receptors. The ventral tegmental area (VTA) contains dopaminergic neurons that play a central role in regulating reward and motivation, projecting to the nucleus accumbens (NAc) and prefrontal cortex (PFC), which together constitute core components of the brain’s reward circuitry (Haber and Knutson, 2010). Multiple preclinical and some clinical investigations have identified 5-HT_2A_ and 5-HT_2C_ receptors within the VTA, NAc, and in the PFC, where 5-HT_2A_ receptors are present at a substantially higher density than 5-HT_2C_ receptors (Ikemoto et al., 2000; Zhang and Stackman, 2015; Borsini et al., 2020). Therefore, trazodone’s therapeutic effect may be mediated through antagonism of receptors located within these regions, which may, in turn, modulate dopaminergic signaling within mesolimbic pathways. In the context of anhedonia, these projection targets particularly the amygdala, PFC, are considered key sites where dysregulation of dopamine transmission contributes to depressive-like behaviors (Gold et al., 2018). On the other hand, it should be noted that the majority of available preclinical data indicate that activation, rather than antagonism, of 5-HT_2A_ receptors facilitates dopamine release, whereas antagonism of 5-HT_2C_ receptors similarly promotes dopaminergic neurotransmission (Di Matteo et al., 2001; Pytka et al., 2016). In support of this mechanism, Balsara et al. demonstrated in rats that trazodone, administered at doses of 5–20 mg/kg, exerts antagonistic activity at both 5-HT_2A_ and 5-HT_2C_ receptors; however, within this dose range, the facilitation of dopaminergic neurotransmission appears to be primarily mediated by 5-HT_2C_ receptor blockade, which mitigates tonic serotonergic inhibition of nigrostriatal dopaminergic neurons (Balsara et al., 2005). Thus, the neurobiological underpinnings of this phenomenon remain complex; nevertheless, further investigations employing methodologically rigorous, well-controlled experimental and clinical designs are warranted to validate these preliminary observations and to delineate, with greater precision, the molecular mechanisms by which antagonism of 5-HT_2A_ and 5-HT_2C_ receptors within reward-related brain regions modulates dopaminergic signaling and ultimately translates into measurable clinical improvement in anhedonia. Conversely, for SSRIs, the anticipated effect size may be attenuated due to their limited efficacy in alleviating anhedonia (Serretti, 2025). In some cases, these agents may even exacerbate hedonic deficits, potentially intensifying symptoms (Serretti, 2023). In the study by McCabe et al., which employed functional magnetic resonance imaging to assess neural responses to various stimuli, citalopram was found to decrease activation within reward-related neural circuits, including the ventral striatum, in response to a chocolate rewarding signal (McCabe et al., 2010). These findings suggest that SSRIs may suppress reward processing, which could partly account for their limited therapeutic efficacy in depressive disorders characterized by anhedonia in certain patients (McCabe et al., 2010).

Evaluation of HAM-A scores over the 12-week observation period showed that both trazodone XR and SSRIs produced comparable clinical improvements during the early phase of treatment (at weeks 2 and 4). However, by week 8, trazodone XR exhibited a gradual yet consistent advantage, with a more sustained trajectory of symptom reduction compared with SSRIs. By week 12, trazodone XR maintained this upward trend, achieving the highest effect sizes observed during the study, while the SSRIs response remained relatively stable, indicating a plateau in therapeutic gains.

In the AIS assessment, trazodone XR demonstrated a consistently stronger impact on insomnia symptoms compared to SSRIs. Superiority was already evident at 2 weeks of the experiment and became more pronounced over time, with trazodone XR showing large effect sizes at each subsequent measurement, while SSRIs produced predominantly small-to-moderate improvements. The effects of trazodone XR observed in the reduction of anxiety and improvement of sleep quality are most likely associated with its high affinity for serotonin receptors and its antagonistic activity at 5-HT_2A_ and 5-HT_2C_ receptors (Fagiolini et al., 2025). In the context of sleep regulation, trazodone’s effects may also be substantially mediated through its interactions with histaminergic and adrenergic pathways (Fagiolini et al., 2023). It has been demonstrated that acute SSRIs administration may induce anxiety, at least in part, via activation of 5-HT_2C_ receptors, highlighting the role of these receptors in the pathophysiology of anxiety (Lin et al., 2023). Regarding SSRIs, it appears that only prolonged administration is likely to result in a clinically meaningful reduction in anxiety symptoms, an effect that may be mediated by the gradual desensitization of 5-HT_2A_ and 5-HT_2C_ receptors over time (Stahl, 2009).

Therefore, given that our study spanned only 12 weeks, it is likely that SSRIs had insufficient time to fully manifest their anxiolytic potential, while trazodone XR exhibited a comparatively faster onset of therapeutic efficacy. Moreover, insomnia, which can persist with continued SSRIs use, may further underscore the potential advantage of trazodone in this context. Consistent with this, a recent meta-analysis found that trazodone increases total sleep time and enhances sleep quality and continuity, while exerting only slight effects on measures like sleep latency and daytime functioning (Kokkali et al., 2024).

This study, as previously noted, was conducted at a single center and employed a naturalistic, open-label design without random allocation. A pooled analysis of different SSRIs may have introduced therapeutic heterogeneity (differences in pharmacodynamics, dosing, and time to onset of action), which could have diluted the true differences compared with trazodone XR and limited the generalizability of the findings to individual compounds. Moreover, fluoxetine—the prototypical SSRI—was not included among the analyzed SSRIs. This was primarily attributable to the naturalistic, observational design of the study: during the recruitment period, none of the patients presented a clinical symptom profile warranting initiation of fluoxetine therapy. Furthermore, pharmacoepidemiological data from Poland indicate that in 2018 sertraline accounted for 38.5% and escitalopram for 21.8% of all SSRI prescriptions, whereas fluoxetine represented only 13.0%, corroborating its comparatively lower current use (Bliźniewska-Kowalska et al., 2020). Similar prescribing trends have been reported in the United Kingdom, where sertraline became the most frequently prescribed antidepressant in the same year (Bogowicz et al., 2021). While our observational study enhances the external validity of the findings by increasing their applicability to real-world clinical settings, it is, conversely, limited by the absence of randomization and blinding—design elements widely regarded as essential for minimizing confounding factors and reducing the risk of bias (Higgins et al., 2024). Furthermore, it should be taken into account that the absence of a control arm, the presence of certain baseline differences, and additional factors primarily dependent on the patient, such as treatment adherence, concomitant therapies, and clinician-specific decision-making within the context of our study, may have additionally affected the internal validity and interpretation of the findings described above.

On the other hand, contemporary research paradigms are shifting, with increasing recognition of the value of incorporating non-randomized interventional studies alongside randomized controlled trials (RCTs) to enhance the strength and applicability of clinical evidence (Yao et al., 2025). This development is driven by the growing recognition that, particularly in psychiatry, evaluating treatment effects in patients is inherently challenging due to the subjective nature of symptoms and the limited availability of objective efficacy markers (Kane, 2002).

This evolving research landscape is increasingly shaped by the complexity of modern therapies, the restrictive eligibility criteria applied in many clinical trials, and the resulting limited target populations. Consequently, the integration of various types of clinical data is gaining increasing recognition across the entire drug development and evaluation continuum, including post-marketing phases (Kennedy-Martin et al., 2015). This perspective supports our findings and underscores their relevance; nonetheless, it should be emphasized that further in-depth analyses, particularly RCTs, are needed to allow for a comprehensive and conclusive interpretation of the aggregated results.

## 5 Conclusion

Trazodone XR demonstrated greater and progressively increasing effect sizes compared to SSRIs, indicating a more robust and sustained antidepressant response. The observed trajectory suggests that trazodone XR may confer benefits extending beyond the reduction of depressive symptoms, encompassing broader affective and behavioral dimensions. Further randomized controlled studies are warranted to confirm these findings and clarify their broader clinical implications.

## Data Availability

The raw data supporting the conclusions of this article will be made available by the authors, without undue reservation.
